# Targeting Myeloperoxidase (MPO) Mediated Oxidative Stress and Inflammation for Reducing Brain Ischemia Injury: Potential Application of Natural Compounds

**DOI:** 10.3389/fphys.2020.00433

**Published:** 2020-05-19

**Authors:** Shuang Chen, Hansen Chen, Qiaohui Du, Jiangang Shen

**Affiliations:** ^1^School of Chinese Medicine, The University of Hong Kong, Pok Fu Lam, Hong Kong; ^2^Shenzhen Institute of Research and Innovation, The University of Hong Kong, Shenzhen, China

**Keywords:** ischemic stroke, myeloperoxidase, natural compound, neuroinflammation, oxidative stress

## Abstract

Oxidative stress and inflammation are two critical pathological processes of cerebral ischemia-reperfusion injury. Myeloperoxidase (MPO) is a critical inflammatory enzyme and therapeutic target triggering both oxidative stress and neuroinflammation in the pathological process of cerebral ischemia-reperfusion injury. MPO is presented in infiltrated neutrophils, activated microglial cells, neurons, and astrocytes in the ischemic brain. Activation of MPO can catalyze the reaction of chloride and H_2_O_2_ to produce HOCl. MPO also mediates oxidative stress by promoting the production of reactive oxygen species (ROS) and reactive nitrogen species (RNS), modulating the polarization and inflammation-related signaling pathways in microglia and neutrophils. MPO can be a therapeutic target for attenuating oxidative damage and neuroinflammation in ischemic stroke. Targeting MPO with inhibitors or gene deficiency significantly reduced brain infarction and improved neurological outcomes. This article discusses the important roles of MPO in mediating oxidative stress and neuroinflammation during cerebral ischemia-reperfusion injury and reviews the current understanding of the underlying mechanisms. Furthermore, we summarize the active compounds from medicinal herbs with potential as MPO inhibitors for anti-oxidative stress and anti-inflammation to attenuate cerebral ischemia-reperfusion injury, and as adjunct therapeutic agents for extending the window of thrombolytic treatment. We highlight that targeting MPO could be a promising strategy for alleviating ischemic brain injury, which merits further translational study.

## Introduction

Stroke is a major human disease burden leading to death and life-long disability. Tissue plasminogen activator (t-PA) is the only therapeutic drug for ischemic stroke approved by the US Food and Drug Administration (FDA). However, t-PA has a restricted time window of 4.5 h, and delayed t-PA infusion increases the risk of hemorrhagic transformation and carries high mortality (Wardlaw et al., 2012). Delayed t-PA infusion mediates cerebral ischemia-reperfusion injury. Thus, it is important to develop novel therapy or/and combination agents for thrombolytic therapy to prevent and attenuate cerebral ischemia-reperfusion injury in ischemic stroke treatment.

The brain has a large oxygen consumption due to high a metabolic rate, and the sudden arrest of oxygen triggers oxidative stress, which plays an important role in mediating cerebral ischemia-reperfusion injury (Allen and Bayraktutan, 2009; Rodrigo et al., 2013). Recanalization with delayed thrombolytic treatment further produces reactive oxygen/nitrogen species (ROS/RNS) such as superoxide (O2^–^), hydrogen peroxide (H_2_O_2_), hypochlorous acid (HOCl), nitric oxide (NO), and peroxynitrite (ONOO^–^). Antioxidant therapies such as edaravone, NXY-059, and allopurinol improved outcomes of acute ischemic stroke patients (Lees et al., 2006; Muir et al., 2008; Nakase et al., 2011). Many other antioxidants also have the potential to reduce cerebral ischemia injury. For example, nicotinamide adenine dinucleotide (NAD +) is a crucial player in modulating cellular energy metabolism and oxidative damage (Kiss et al., 2019a, b). Cellular NAD + level was significantly decreased during cerebral ischemia/reperfusion injury and exogenous NAD + supplementation prevented oxidative stress and increased the production of ATP, subsequently reducing cerebral ischemia/reperfusion injury (Ying et al., 2007; Huang Q. et al., 2018). Recently, the roles of RNS, including nitrogen monoxide, nitrogen dioxide, and peroxynitrite, etc., have drawn significant attention from the scientific community. RNS could activate multiple cellular signaling pathways involved in the BBB disruption, infarction enlargement and apoptotic cell death in cerebral ischemia-reperfusion injury (Chen et al., 2013). Matrix metalloproteinase (MMP), a classic proteolytic enzyme, is one of the critical molecular targets of RNS in mediating neuroinflammation and hemorrhagic transformation during cerebral ischemia-reperfusion injury (Gasche et al., 1999; Yang Y. et al., 2007; Jickling et al., 2014; Vandooren et al., 2014). MMP-9 is a plasma biomarker for monitoring brain damage and predicting hemorrhagic transformation in ischemic stroke with thrombolytic treatment (Ramos-Fernandez et al., 2011). *N*G-nitro-*L*-arginine methyl ester (*L*-NAME), a non-selective NOS inhibitor, attenuated the BBB disruption through inhibiting MMP-9 activity in an experimental ischemic stroke animal model (Gursoy-Ozdemir et al., 2004). For decades, we have made great efforts to explore the roles of RNS in mediating BBB disruption and brain injury, and seeking therapeutic targets for drug discovery to attenuate cerebral ischemia-reperfusion injury (Shen et al., 2006; Liu et al., 2009; Gu et al., 2012; Chen et al., 2013, 2019; Chen H. et al., 2018; Chen H.S. et al., 2015; Fu et al., 2014; Gong et al., 2015; Feng et al., 2018a, b). With the short lifetime of ROS and RNS, it is an extremely challenging task to directly detect ROS/RNS in viable systems. To resolve the problems, we have successfully developed several fluorescent probes with high specificity and sensitivity, and applied the probes to detect peroxynitrite and hypochlorous acid in ischemic stroke rat models (Peng et al., 2014, 2016; Bai et al., 2020). We found that the RNS production aggravates the BBB disruption and brain damage in cerebral ischemia-reperfusion injury through modulating caveolin-1 and MMPs, and targeting RNS/caveolin-1/MMPs signaling cascades could be an important therapeutic strategy to attenuate cerebral ischemia-reperfusion injury (Chen H.S. et al., 2015, Chen H. et al., 2018; Chen H.S. et al., 2018). Treatment of peroxynitrite scavengers attenuated the BBB damage, neuronal cell death and hemorrhagic transformation in different experimental ischemic stroke models (Xu et al., 2013; Chen H. et al., 2018; Chen et al., 2019). In our recent review articles, we highlight peroxynitrite as a therapeutic target for ischemic stroke (Chen et al., 2013, 2016; Chen H.S. et al., 2018).

Inflammatory factors play crucial roles in cerebral ischemia-reperfusion injury. At the early stage of cerebral ischemia, the activation of glial cells and infiltration of leukocytes in the injured tissue produced great amounts of pro-inflammatory factors such as TNF-α and IL-1β, contributing to BBB damage and hemorrhagic transformation (Jayaraj et al., 2019; Yang C. et al., 2019). The infiltrated neutrophils produced inflammatory factors and aggravated cerebral ischemia-reperfusion injury (Anrather and Iadecola, 2016). Furthermore, reactive and proliferative astrocytes can also disrupt neurovascular cell balance and form a neuroinflammatory environment that is harmful to neurogenesis (Popa-Wagner et al., 2019). Inflammatory factors can activate MMPs, disrupt BBB integrity and worsen brain damage (Rosell et al., 2008; Jickling et al., 2015). The cross-talk between oxidative stress and neuroinflammation in ischemic stroke has been extensively studied (Collino et al., 2006; Chehaibi et al., 2016; Reiche et al., 2019; Yang Q. et al., 2019; Zhou F. et al., 2019). ROS activates various inflammatory factors for inducing neural cell death, disrupting the integrity of the BBB and enlarging the infarct volume (Crack and Taylor, 2005; Chen H.S. et al., 2018). The production of ROS could aggravate inflammatory responses of the peripheral immunological system and central nervous system by inducing the activation of adhesion molecules and promoting immunocyte infiltration (Granger and Kvietys, 2015; Mizuma and Yenari, 2017; Chen H.S. et al., 2018; Sun et al., 2018). The cross-talk and interaction of ROS/RNS and inflammatory factors could be a critical pathological mechanism and therapeutic target in cerebral ischemia-reperfusion injury. In this review article, we focus on the roles of critical neuroinflammatory enzyme myeloperoxidase (MPO) in mediating oxidative stress and neuroinflammation in cerebral ischemia-reperfusion injury, and summarize recent progress made in bioactive compounds from medicinal plants with antioxidant and anti-inflammation effects for ischemic stroke treatment.

## Roles of MPO in Ischemic Brain Injury by Mediating Inflammation and Oxidative Stress

As a heme-containing peroxidase, MPO is highly expressed in multiple inflammatory cells, including neutrophils, activated microglia, monocytes/macrophage, as well as astrocytes and neurons (Nagra et al., 1997; Green et al., 2004; Forghani et al., 2015; Yu et al., 2016). MPO genetic variability appears to increase the risk of ischemic stroke (Manso et al., 2011) and MPO polymorphisms could be associated with the severity of brain damage and functional outcomes (Hoy et al., 2003). The increased MPO activity was reported in both experimental stroke animal models and ischemic stroke patients (Barone et al., 1991; Cojocaru et al., 2010; Kong et al., 2014; Jin et al., 2018). The MPO activity in the ischemic cortex was increased early at 6 h of ischemia onset, peaked at day 5, and gradually returned to basal level at day 15 in both transient middle cerebral artery occlusion (tMCAO) and permanent MCAO (pMCAO) animal models (Barone et al., 1995). Interestingly, tMCAO had a significantly higher MPO level in the ischemic cortex than the pMCAO model after stroke onset (Barone et al., 1995), indicating that reperfusion could aggravate the activation of MPO for neuroinflammation. The penumbra area had higher MPO expression than the core (Horvath et al., 2018). With the survival of neural cells in the penumbra, this discovery raises the potential therapeutic value to prevent further loss of neural cells in the ischemic brain. Clinical studies yield similar results to animal experiments. The increase of MPO level in plasma was associated with the severity of ischemic brain damage in ischemic stroke patients (Palm et al., 2018; Tziomalos et al., 2019). A functional MRI study reported the positive correlation between MPO expression and infarct volume in ischemic stroke rat brains (Breckwoldt et al., 2008).

The neutrophil activation and degranulation appear to be important mediators for MPO induction and the source of MPO in plasma (Tay et al., 2015). MPO activity derived from neutrophils was peaked at day 1–3 of stroke onset, whereas MPO from the macrophage/microglia at day 5–7 (Breckwoldt et al., 2008). Neutrophil inhibitory factor (rNIF) was revealed to reduce neutrophil infiltration and infarct size in the ischemic brain (Barone et al., 1995). Neutrophil depletion by using an anti-neutrophil monoclonal antibody (RP3) completely inhibited MPO activity, attenuated brain edema and reduced brain infarction in the ischemic brain after 24 h of reperfusion (Matsuo et al., 1994). Thus, neutrophil-mediated MPO activation contributes to inflammation and the severity of brain damage during ischemic stroke.

Myeloperoxidase activation also plays crucial roles in oxidative damage in ischemic stroke. N-acetyl lysyltyro sylcysteine amide (KYC, an MPO inhibitor) attenuated oxidative and nitrative damage in the cortex of ischemic core (Yu et al., 2016). Hypochlorous acid (HOCl) is a crucial cytotoxic factor contributing to the MPO-mediated oxidative injury in ischemic stroke. Activated MPO induces HOCl production *via* catalyzing the reaction of chloride and H_2_O_2_ to induce chlorinative stress (Weiss et al., 1982; Marquez and Dunford, 1994; Yap et al., 2007). HOCl has high diffusivity and oxidative activity to react with lipids, proteins and DNA (Schraufstatter et al., 1990; Prutz, 1996; Panasenko, 1997; Hawkins et al., 2003; Pattison et al., 2003). Activated phagocytes produce HOCl and recruit inflammatory cells to ischemic brain regions, subsequently mediating the BBB damage (Ullen et al., 2013). Of note, HOCl itself can exacerbate oxidative stress, promote the translocation of p67(phox) and p47(phox) of NAD(P)H oxidase and mediate the production of superoxide, peroxynitrite and oxidized eNOS dimer in endothelial cells (Xu et al., 2006). Genetic deletion or pharmacological intervention with MPO inhibitors decreased inflammatory cell recruitment, reduced infarct volume, protected the BBB integrity, attenuated neurological deficit and improved survival rates in rodent ischemic stroke model (Forghani et al., 2015; Yu et al., 2016; Kim et al., 2019). The MPO inhibition with 4-aminobenzoic acid hydrazide (ABAH) or MPO deficiency may create a protective environment that decreases inflammatory cell recruitment and increases survival factors to improve functional outcome (Kim et al., 2019). Of note, the MPO inhibitor was more effective when treated at the subacute phase than the acute phase (Forghani et al., 2015). The robust protection of the MPO inhibitor at the subacute phase was consistent with the delayed peak of MPO expression in the ischemic brain (Barone et al., 1995; Breckwoldt et al., 2008). These studies indicate that the MPO-mediated inflammation at the subacute phase could be a critical underlying mechanism contributing to inflammatory brain damage in ischemic stroke. Importantly, MPO inhibition may represent a promising therapeutic target for stroke therapy, particularly even days after the stroke has occurred. Given the reality that most stroke patients cannot make the golden therapeutic window for thrombolysis, further investigations in this aspect may create a novel therapeutic window for improving the outcome of ischemic stroke by reducing the MPO-mediated inflammation and oxidative injury practically. Therefore, the MPO-mediated oxidative stress and neuroinflammation could be critical therapeutic targets for reducing ischemic brain injury.

Furthermore, the MPO-mediated inflammation affects post-stroke neurogenesis. Treatment of 4-ABAH promoted neurogenesis, and induced proliferation of astrocytes in the subventricular zone (SVZ), striatum and cortex (Kim et al., 2016). MPO knockout mice had increased cell proliferation and improved neurological outcomes in post-ischemic stroke rats (Kim et al., 2016). MPO inhibitor KYC decreased the pro-inflammatory M1 microglial cells and N1 neutrophils, increased the proliferation and differentiation of neuronal stem cells in the ischemic cortex, and protected the exogenous neural stem cells in the ischemic brain (Yu et al., 2018). Therefore, MPO exerts its roles in mediating oxidative stress and inflammation and affects adult neurogenesis in the post-stroke brain.

The MPO-mediated neuroinflammation involves multiple cellular mediators and signaling pathways. PI3K/AKT signaling is one of the cellular signaling pathways in the MPO-mediated inflammation during ischemic brain injury. LY294002, a PI3K/AKT inhibitor, abolished the effects of 5-LOX inhibitor Zileuton on inhibiting MPO activity in ischemic brain injury (Tu et al., 2016). LY294002 eliminated the neuroprotective effects of repetitive ischemic preconditioning and its underlying mechanisms could be related to regulating MPO activity (Tu et al., 2015). Except for PI3K/Akt pathway, ADAMTS13 (a disintegrin and metalloprotease with thrombospondin type I repeats-13) can inhibit MPO activity by inactivating the hyperactive ultra-large von Willebrand factor (ULVWF). The MPO activity was enhanced in ADAMTS13-deficient mice but was reduced in VWF-deficient mice under focal cerebral ischemia (Khan et al., 2012). In addition, E-selectin deficient mice showed the reduction of MPO expression in the ischemic brain, possibly *via* reducing the neutrophil infiltration (Ma et al., 2012). PARP also regulates neutrophil infiltration and MPO activity. The PARP inhibitor 3-aminobenzamide (3-AB) largely decreased MPO activity in the ischemic brain (Couturier et al., 2003). Therefore, MPO can be modulated by multiple cellular signaling mechanisms, and MPO is one of the inflammatory factors contributing to the pathology of ischemic stroke through a complex interaction with different cellular signaling molecules, which remains to be further elucidated.

## MPO Activation and Thrombolysis-Induced Ischemic Brain Injury

Inflammatory factors mediate hemorrhage transformation in ischemic stroke with delayed t-PA treatment. Anti-leukocyte adhesion antibody (anti-CD18) significantly decreased the neurological deficit in a rat ischemic stroke model with t-PA treatment, indicating the potential of targeting leukocytes to extend the therapeutic window of t-PA (Barone et al., 1995). The t-PA treatment increased the MPO level in the plasma of ischemic stroke patients within 1 h (Dominguez et al., 2010). Notably, MPO activation can trigger oxidative stress and nitrosative stress in the ischemic brain by forming the chlorotyrosine and nitrotyrosine (Yu et al., 2016). We recently reported that nitrotyrosine was associated with MMPs activation, BBB disruption and hemorrhage transformation in ischemic stroke with delayed t-PA treatment (Chen H.S. et al., 2015; Chen H. et al., 2018). The MPO-mediated nitrosative stress could be a potential player in mediating hemorrhagic transformation in ischemic stroke with the delayed t-PA treatment. Notably, treatment of taurine, a HOCl scavenger, reduced the rates of hemorrhage transformation in experimental ischemic stroke animal model with delayed t-PA treatment (Guan et al., 2011). Hence, the production of HOCl might be a crucial cytotoxic factor in the MPO-mediated oxidative injury and inflammation. Inhibition of MPO-HOCl is a potential therapeutic strategy to minimize hemorrhage transformation, which warrants further investigation.

In summary, recent progress indicates that the study on the MPO-mediated oxidative injury and inflammation not only brings novel insight into understanding the molecular pathology of ischemic brain injury but also represents a promising strategy for drug discovery to target MPO for improving stroke outcome.

## Medicinal Herbal Compounds With the MPO-Inhibiting Activity Showing Antioxidant, Anti-Inflammation, and Neuroprotective Effects

**FIGURE 1:**
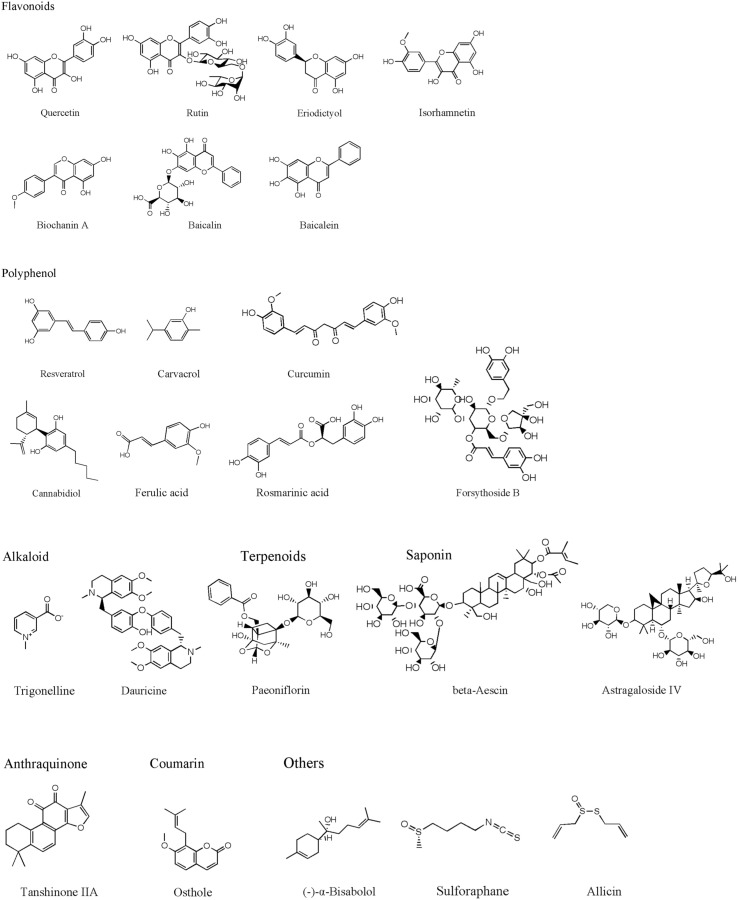
The chemical structures of the natural compounds.

Traditional herbal medicine is an important source for drug discovery due to its long history in clinical practice. Recent studies identified many active compounds with the bioactivities of inhibiting MPO activity, which can be potentially used to attenuate cerebral ischemia-reperfusion injury. Herein, we summarize representative active compounds in the following session. The chemical structures of the compounds refer to Figure 1.

## Flavonoids

Bioflavonoids are well-known antioxidants with neuroprotective properties. Quercetin is a representative flavonoid compound widely existed in medicinal herbs, including Ginkgo biloba leaves and others (Wu X.J. et al., 2019). Quercetin revealed its bioactivities of anti-oxidation, anti-tumor, anti-inflammatory, anti-platelet aggregation, etc. (Mosawy et al., 2013; Li Y. et al., 2016; Chen Z. et al., 2018; Wu L. et al., 2019). Treatment of quercetin (30, 50 mg/kg) attenuated neuronal cell death and reduced infarct size in both global and focal cerebral ischemia animal models (Ahmad et al., 2011; Annapurna et al., 2013; Park et al., 2018). Quercetin inhibited MPO activity and reduced oxidative stress-mediated neural damage in ischemic brain tissues (Annapurna et al., 2013). Rutin is another common and wild distributed flavonoid extracted from medicinal plants, such as Mulberry (Morus alba L.) (Zhao et al., 2015). Pre-treatment with rutin for 21 days (25 mg/kg, daily) significantly attenuated brain damage through up-regulating antioxidant enzymes and inhibiting oxidative stress (Khan et al., 2009). Rutin also revealed to inhibit MMP-9 activity and reduce BBB disruption (Jang et al., 2014). Interestingly, its neuroprotective and antioxidant effects are related to inhibiting MPO activity (Annapurna et al., 2013).

Eriodictyol is widely distributed in fruits and vegetables, and isolated from the Chinese herb Dracocephalum rupestre. Oral administration of eriodictyol (1, 2, 4 mg/kg) exhibited inhibitory effects on MPO expression and inflammation in a permanent ischemic stroke rat model. Eriodictyol treatment down-regulated the expression of TNF-α and iNOS in the ischemic cortex, decreased infarct size, improved motor function, and attenuated the memory deficit in permanent ischemic stroke rats (Ferreira Ede et al., 2016). Eriodictyol-7-O-glucoside (30 mg/kg), also showed neuroprotective effects against cerebral ischemic injury through activating the antioxidant signaling nuclear factor erythroid-2-related factor 2/antioxidant response element (Nrf2/ARE) (Jing et al., 2013).

Isorhamnetin is a typical flavonoid extracted from *Hippophae rhamnoides* (Li et al., 2015). Treatment of isorhamnetin (5 mg/kg) suppressed MPO activity, attenuated oxidative/nitrosative stress, inhibited inflammatory factors (IL-1β, IL-6, and TNF-α), preserved tight junction proteins and protected the BBB integrity in the acute ischemic stroke model (Zhao et al., 2016). The anti-inflammation and anti-oxidative bioactivities of isorhamnetin were also found in the *in vitro* cultured brain microvascular endothelial cells under oxygen and glucose deprivation (OGD) condition (Li W. et al., 2016).

Biochanin A, an O-methylated natural isoflavonoid, mainly exists in red clover, chickpea or other legumes. Biochanin A revealed various pharmacological functions, including antioxidation, anti-inflammation and anti-tumorigenesis (Mishra et al., 2008; Liu et al., 2016; Guo et al., 2019). Biochanin A suppressed the activity of MPO and down-regulated the expression of IL-1β and TNF-α in acute ischemic brain injury. Treatment with biochanin A (10, 20, 40 mg/kg) significantly improved neurological outcomes, reduced infarct volume and brain edema in post-ischemic brain injury (Wang et al., 2015).

Baicalin and its aglycon baicalein are flavonoids extracted from *Scutellaria baicalensis*, a medicinal plant. Both baicalin and baicalein revealed their neuroprotective effects against cerebral ischemia-reperfusion injury and the underlying mechanisms were related to their anti-oxidative stress, anti-apoptotic, anti-inflammation and anti-excitotoxicity properties (Tu et al., 2009, 2011a,b; Cui et al., 2010; Xue et al., 2010; Li H.Y. et al., 2012; Li et al., 2017; Liang et al., 2017; Yang et al., 2019). Baicalin (30, 100 mg/kg) and baicalein (50 mg/kg) showed to inhibit the MPO activity in the ischemic brain injury (Hwang et al., 2002; Tu et al., 2009). Our recent studies suggest that baicalin reduced infarct size, minimized the BBB damage and hemorrhage transformation in the experimental ischemic stroke model with delayed t-PA treatment. The underlying mechanisms could be attributed to the direct peroxynitrite-scavenging property (Xu et al., 2013; Chen H. et al., 2018).

## Polyphenol

Resveratrol is a polyphenolic compound with a terpenoid structure, mainly found in plants such as grapes, peanuts, mulberry and knotweed. Resveratrol has been used for aging-related diseases such as cancer, cardiovascular diseases and stroke (Liu et al., 2013, 2018; Dou et al., 2019; Zhou X. et al., 2019). Resveratrol treatment (50 mg/kg) has potent anti-oxidative stress and anti-inflammatory properties and has neuroprotective effects against acute ischemic brain injury (Dou et al., 2019). The neuroprotective mechanisms involve multiple molecular targets such as MMP-9 (Pandey et al., 2015; Wei et al., 2015), NMDA receptor-mediated ERK-CREB signaling pathway (Li W. et al., 2016), JAK2/STAT3 pathway (Hou et al., 2018), neurotransmitters and neuromodulators, excitatory neurotransmitter glutamates, aspartate and neuromodulator d-Serine, etc. (Li et al., 2010). The inhibitory effects of resveratrol on MPO activity contributed to its neuroprotective effects (Fang et al., 2015; Lei and Chen, 2018; Lei et al., 2019). Furthermore, resveratrol had the capacity to cross BBB (Wang et al., 2002) and fusogenic liposomes could enhance the delivering efficiency of resveratrol across the BBB (Wiedenhoeft et al., 2019). Carvacrol is a monoterpene phenol and an isomer of thymol commonly found in many aromatic plants including *Origanum dictamnus, Origanum vulgare*, and *Origanum majorana*. Carvacrol treatment attenuated neuronal apoptosis, reduced infarct size and improved the neurological outcomes in both adult focal ischemic stroke model and neonatal cerebral hypoxic-ischemic model (Yu et al., 2012; Chen W. et al., 2015). Furthermore, carvacrol (10, 20, 40 mg/kg) dose-dependently down-regulated the MPO activity and the expressions of iNOS and COX-2 in the ischemic brain (Li Y. et al., 2016).

Curcumin is a polyphenol in the curry spice turmeric. In ancient Chinese and Indian medicine, curcumin was used to treat various diseases. Curcumin has multiple molecular targets, including transcription factors, growth factors and their receptors (Kunnumakkara et al., 2017). Curcumin has revealed its anti-inflammatory, antioxidant, anti-tumor and cardiovascular protective properties (Menon and Sudheer, 2007; Jiang et al., 2017; Kunnumakkara et al., 2017). The neuroprotective effects of curcumin treatment (150, 200 mg/kg) are related to inhibiting leukocyte infiltration, regulating microglia/macrophage polarization and inflammatory factors production, and inhibiting autophagy against cerebral ischemia-reperfusion injury (Liu et al., 2017; Huang L. et al., 2018; Bavarsad et al., 2019). The neuroprotective effects of curcumin are related to inhibition of TLR2/4-NF-κB signaling pathway and reduction of MPO activity in the ischemic brain (Tu et al., 2014).

Cannabidiol (CBD) isolated from *Cannabis sativa L. (Cannabaceae)* (Brand and Zhao, 2017) was reported to increase the cortex blood flow *via* the serotonergic 5-hydroxytryptamine 1A receptor-dependent mechanism (Mishima et al., 2005). CBD treatment (3 mg/kg) attenuated neuroinflammation through inhibiting MPO and HMGB1 in the ischemic brain (Hayakawa et al., 2008). A systematic review supported the neuroprotective effects of CBD in ischemic stroke treatment (England et al., 2015). The neuroprotective mechanisms were also related to inhibition of inflammatory factors NF-κB and tumor necrosis factor receptor 1 (TNFR1) (Khaksar and Bigdeli, 2017).

*Angelica sinen-sis (Oliv.) Diels (AS)* and *Ligusticum chuanxiong Hort. (LC)* are medicinal herbs used for stroke treatment for centuries in China. Ferulic acid (FA) is a bioactive ingredient of AS and LC. FA has revealed to scavenge superoxide radicals and restore vasodilation in spontaneously hypertensive rats (Suzuki et al., 2007). FA treatment at 100 mg/kg at the beginning of the MCAO stroke model effectively reduced infarct size and improved neurological deficits. The underlying mechanisms were related to inhibiting ICAM-1 and NF-κB, and reducing the infiltrations of the MPO immune-reactive cells (Cheng et al., 2008). Rosmarinic acid (RA) is a natural phenolic compound isolated from Chinese herb *Salvia miltiorrhiza* (Wu et al., 2015). RA treatment (50 mg/kg) inhibited HMGB1 expression and NF-κB activation, reduced the BBB permeability, decreased infarct size and alleviated neurological deficits in cerebral ischemia-reperfusion injury (Luan et al., 2013). The inhibitory effects of RA on MPO contributed to its neuroprotective effects against ischemic brain injury (Fonteles et al., 2016).

Forsythoside B is an anti-inflammatory compound extracted from the leave of *Lamiophlomis rotata Kudo*. Even delayed administration of forsythoside B at 5 h after reperfusion had a neuroprotective effect. Forsythoside B at the dosage of 20 mg/kg attenuated brain infarct size, brain edema, and BBB permeability through inhibiting MPO activity and NF-κB expression against cerebral ischemia/reperfusion injury (Jiang et al., 2010).

## Alkaloids

Alkaloids are nitrogen-containing alkaline compounds from medicinal herbs. Trigonelline and dauricine are representative alkaloid with the MPO inhibitory effects. Intraperitoneal injection of trigonelline (100 mg/kg), a plant alkaloid from fenugreek seeds, inhibited MPO expression, improved the neurological outcomes and alleviated infarct size in ischemic stroke animal models (Pravalika et al., 2019). Dauricine is a BBB permeable bisbenzyl isoquinoline alkaloid extracted from *menispermum dauricum DC* root. Dauricine treatment at 5 or 10 mg/kg revealed its neuroprotective effects and the underlying mechanisms were associated with the suppression of TNF-α, IL-1β, and ICAM-1 expression and inhibition of PMNs infiltration (Yang X.Y. et al., 2007).

## Terpenoids

Paeoniflorin is a natural component derived from *Paeonia lactiflora Pall* and has anti-inflammatory properties. Intravenous injection of paeoniflorin (10,15, 20 mg/kg) at 10 min before or 30 min after MCAO effectively reduced infarct size and ameliorated the neurological deficit via inhibiting PMN infiltration and down-regulating inflammatory factors including TNF-α, IL-1β, and ICAM-1 (Tang N.Y. et al., 2010).

## Saponins

Saponins are widely distributed in various plant species. As the active components in many medicinal herbs, saponins possess diverse biological activities, such as anti-inflammatory, anti-oxidative stress and neuroprotective effects (Guclu-Ustundag and Mazza, 2007). β-Aescin is a main bioactive compound isolated from *Aesculus hippocastanum*, a commonly used medicinal herb for vascular disorders in Traditional Chinese Medicine. Aescin has anti-inflammatory, anti-edema and anti-oxidation effects (Cheng et al., 2016; Gallelli, 2019). Pretreatment of β-Aescin (15, 30, 60 mg/kg) for 7 days significantly inhibited the MPO activity, reduced the neutrophils migration, lessened infarct sizes, ameliorated neurological deficit in the rat model of MCAO cerebral ischemia-reperfusion injury (Hu et al., 2004). Astragaloside IV, a major component of *Astragalus membranaceus*, showed its anti-inflammatory property in the focal cerebral ischemia-reperfusion rat model. Treatment of Astragaloside IV (10, 20 mg/kg) at the onset of ischemia or at 12 h after the reperfusion significantly alleviated infarct volume and neurological deficit. The underlying mechanisms could be related to inhibiting MPO, TNF-α, IL-1β, NF-κB expression and neutrophil adhesion (Li M. et al., 2012).

## Polysaccharides

Fucoidan is a sulfated polysaccharide mainly exited in brown algae and brown seaweed. Treatment with fucoidan (80, 160 mg/kg) inhibited MPO activity and inflammation-associated cytokines such as IL-1, IL-6, TNF-α, and attenuated cerebral ischemia-reperfusion injury (Che et al., 2017).

## Anthraquinone

Tanshinone IIA is a key bioactive compound in *Salvia miltiorrhiza*, a commonly used medicinal herb for cardiovascular and cerebral vascular diseases. Tanshinone IIA had neuroprotective effects against focal cerebral I/R injury, and its underlying mechanisms were considered to inhibit the expression of NF-κB, MMP-9 and HMGB1 (Liu et al., 2010; Tang C. et al., 2010). Meanwhile, Tanshinone IIA (25 mg/kg) also revealed to inhibit MPO activity, attenuate macrophage migration inhibitory factor, TNF-α and IL-6, and ameliorate BBB permeability and neurological dysfunction (Chen et al., 2012).

## Coumarin

Osthole, a natural coumarin derivative, is a bioactive compound from many medicinal herbs such as *Angelica pubescens, Cnidium monnieri*, and *Peucedanum ostruthium*. Osthole was reported to improve chronic cerebral hypoperfusion induced cognitive deficits and neuronal damage in the hippocampus (Ji et al., 2010). Osthole revealed its bioactivities of inhibiting MPO, MMP-9, IL-1β, and IL-8 for the neuroprotective effects against cerebral ischemic injury (Chao et al., 2010; Mao et al., 2011).

## Other Compounds

Other compounds, like (-)-α-bisabolol, sulforaphane, and allicin, also have anti-inflammation effects on inhibiting inflammatory cytokines, including TNF-α, IL-1β, and IL-6. Those compounds are considered as potential candidates for adjuvant therapy against cerebral ischemia-reperfusion injury. For example, (-)-α-bisabolol is an unsaturated sesquiterpene alcohol existed in a variety of plants, such as *Matricaria chamomilla, Salvia runcinata* and *Myoporum crassifolium*. (-)-α-bisabolol has anti-inflammatory, antioxidant and anti-apoptotic activities (Sampaio et al., 2016). (-)-α-bisabolol (200 mg/kg) showed its neuroprotective effects on reducing infarct size and neurological deficits in a permanent MCAO animal model via inhibiting MPO, TNF-α and iNOS (Fernandes et al., 2019). Sulforaphane, an isothiocyanate occurring in cruciferous vegetables, transcriptionally up-regulated the genes controlling aerobic cells and inhibited oxidative stress and inflammation (Zhang et al., 1992). Intraperitoneally treatment with sulforaphane (10 mg/kg) at the onset of reperfusion dramatically ameliorated infarct volume, alleviated neurological deficit, and decreased neutrophils infiltration in ischemic brain injury. Sulforaphane also down-regulated the expressions of cleaved caspase-1, IL-1β, and IL-18 and inhibited the activation of NLRP3 inflammasome (Yu et al., 2017). Allicin is a major active compound in garlic. Allicin has anti-inflammatory, anti-fungal, antioxidant and anti-tumoral activities (Hunter et al., 2005; Chan et al., 2014; Zhou et al., 2014). Allicin treatment (50 mg/kg) revealed to reduce TNF-α level and MPO activity, ameliorated infarct size, alleviated brain edema and improved the neurological score in the experimental MCAO ischemic stroke rat models (Zhang et al., 2015).

## Perspectives

Targeting MPO and cellular signaling cascades could be a therapeutic strategy to decrease infarct size and improve the neurological outcomes in ischemic stroke treatment (Table 1). As an inflammatory enzyme, MPO activation results in HOCl production, increases pro-inflammatory cytokine and mediates protein nitration (Nussbaum et al., 2013). MPO is also a highly versatile oxidative enzyme and participates in the pathological process of oxidative and nitrosative stress (Davies et al., 2008). The crosstalk of oxidative stress and inflammation could amplify brain damage in cerebral ischemia-reperfusion injury. The inhibition of MPO could reach two goals for antioxidant and anti-inflammation simultaneously. Importantly, MPO could be an important molecular target for ischemic stroke treatment practically that allows for a broad intervention time window (Kim et al., 2018). Thus, MPO inhibitors have translational values as therapeutic candidates for improving the outcomes of ischemic stroke treatment.

**TABLE 1:** Intervention of inhibiting MPO for protecting ischemic stroke injury.

Treatment agent	Direct target	Experimental Model	Treatment time point and path	Dosage	Molecules Changes	Major Results	References
4-ABAH	MPO	tMCAO (I 30 min/R 21 day) (Mice)	Daily injections starting on day 2 after MCAO, continuing to day 21. I.P.	40 mg/kg	MPO	Infarct Volume, Neurological deficit, Survival	Forghani et al., 2015
N-acetyl lysyltyrosylcysteine amide (KYC)	MPO	tMCAO (I 30 min/R 3 day) (Mice)	1 h after reperfusion. I.P.	10 mg/kg	NIMP-R14, p53, nNOS, MPO, NO_2_Tyr, 4-HNE	Neurological deficit, Infarct volume, BBB permeability	Yu et al., 2016
4-ABAH	MPO	tMCAO (I 30 min/R 7 day) (Mice)	Daily injections starting on day 2, continuing to day 7 after MCAO. I.P.	40 mg/kg	Hsp70, p-Akt, p53	Neuronal death, Grid walk, Adhesive	Kim et al., 2019
4-ABAH	MPO	tMCAO (I 30 min/R 7 day) (Mice)	at 8 h after the stroke and then twice daily up to day 7. I.P.	40 mg/kg	BDNF, p-CREB, AcH3	Cell proliferation	Kim et al., 2016
N-acetyl lysyltyrosylcysteine amide (KYC)	MPO	tMCAO (I 30 min/R 7 day) (Mice)	starting from 1 h, continuing to 7 days after MCAO. I.P.	10 mg/kg	SOX2, NO_2_Tyr, CITyr	Neurological deficit, brain atrophy, apoptosis	Yu et al., 2018
		tMCAO (I 30 min/R 3 day) (Mice)	starting from 1 h, continuing to 3 days after MCAO. I.P.	10 mg/kg	HMGB1, RAGE, NF-kB, CITyr, NO_2_Tyr, β-catenin	Apoptosis	
							

Both oxidative stress and inflammation are crucial pathological mechanisms in the BBB damage and hemorrhagic transformation in ischemic stroke with delayed t-PA treatment. The increased MPO in the plasma was found in ischemic stroke patients after receiving t-PA treatment (Dominguez et al., 2010). Treatment of MPO inhibitor KYC restored the BBB function in experimental autoimmune encephalomyelitis mice (Zhang et al., 2016), showing its potential to prevent inflammatory factors-mediated BBB damage. Thus, MPO-mediated HOCl production could be a crucial target for promoting anti-oxidative stress and anti-inflammation and preventing hemorrhagic transformation. Developing MPO inhibitors is a promising strategy for expending the golden therapeutic window for t-PA treatment.

Compounds from medicinal herbs are important sources for drug discovery. Many of them already show their potential as drug candidates for ischemic stroke treatment. We provide MPO inhibitory compounds in Table 2. Although many studies provide exciting results about the bioactivities of those compounds on MPO inhibition and neuroprotection in different experimental model systems, we should note that most of the studies only examine the activity and expression of MPO in the ischemic brain *in vivo*. It remains unknown whether those compounds have direct and specific actions of binding MPO or lead molecular modifications to MPO. Since most of the compounds exert antioxidant effects (Table 2), the antioxidant effects would, in turn, inhibit MPO activity in the ischemic brain. It is valuable to examine whether these compounds could directly inhibit MPO or not. On the other hand, the pharmacological effects of those natural compounds may also be due to modulating multiple targets and signaling pathways in the ischemic brain, rather than just a single target (Chen et al., 2017). The one-compound-multi-target pattern may allow for inhibiting MPO activity as well as other inflammatory factors. Furthermore, we would remark that those neuroprotective compounds may serve to reduce the complications of delayed t-PA treatment and extend the therapeutic time window of t-PA. Studies seldomly provide information on pharmacokinetic and pharmacodynamic parameters when the pharmacological studies were performed to explore the neuroprotection of a selected compound. For the translational study, we should also pay specific attention to the pharmacokinetics and pharmacodynamics of those compounds and the toxicological evaluation for further development as therapeutic agents. Moreover, the potential interactions of those compounds with t-PA should be examined before being used as combination therapy.

**TABLE 2:** Natural compound inhibiting MPO for protecting ischemic stroke injury.

Compound	Representative sources	Experimental model	Treatment time point and path	Dosage	targets	Major results	References
Quercetin	Ruta graveolens L.	Global cerebral ischemia (I 30 min/R 4 h)	10 min before reperfusion (not given)	50 mg/kg	MDA, MPO, SOD, CAT	infarct volume	Annapurna et al., 2013
Rutin	Ruta graveolens L.	Global cerebral ischemia (I 30 min/R 4 h)	10 min before reperfusion (not given)	10 mg/kg	MDA, MPO, SOD, CAT	infarct volume	Annapurna et al., 2013
Eriodictyol	Dracocephalum rupestre and citrus fruits	pMCAO	30 mins before and 2 h after pMCAO and lasting for 5 days. I.G.	4 mg/kg	MPO, TNF-α, iNOS	neurological deficit, infarct volume, Open-field test, Y-maze test, Passive avoidance test	Ferreira Ede et al., 2016
Isorhamnetin	Hippophae rhamnoides L., Oenanthe javanica and Ginkgo biloba L.,	tMCAO (I 1 h/R 48 h)	Onset and 24 h of reperfusion. I.P.	5 mg/kg	Caspase-3, occluding, ZO-1, claudin-5, AQP4, MDA, iNOS, Nrf2, HO-1, 3-NT, NMDA NR1, MPO, TNF-α, IL-1β, IL-6	neurological deficit, infarct volume, brain edema, BBB permeability	Zhao et al., 2016
Biochanin A	red clover, chickpea or other legumes	tMCAO (I 2 h/R 24 h)	Lasting for 14 days before MCAO. I.P.	20, 40 mg/kg	MPO, TNF-α, IL-1β, p38	neurological deficit, infarct volume, brain edema,	Wang et al., 2015
Baicalin	Scutellaria baicalensis Georgi	pMCAO (24 h)	2 and 12 h after MCAO. I.P.	30, 100 mg/kg	MPO, iNOS, COX-2, Caspase-3	neurological deficit, infarct volume	Tu et al., 2009
Baicalein	Scutellariae radix	tMCAO (I 2 h/R 22 h)	promptly prior to and 2 h after the reperfusion	50 mg/kg	MPO	infarct volume	Hwang et al., 2002
Resveratrol	grapes, cranberries and peanuts	pMCAO (24 h)	Onset of MCAO. I.P.	100 mg/kg	IL-1β, TNF-α, COX2, MPO, Akt	neurological deficit, brain edema	Lei and Chen, 2018
Resveratrol	grapes, cranberries and peanuts	pMCAO (24 h)	2 h after MCAO. I.P.	100 mg/kg	MPO, TLR4, NF-κB, p65, COX-2, MMP-9, TNF-α, IL-1β	neurological deficit, infarct volume, brain edema, BBB permeability	Lei et al., 2019
Resveratrol	grapes, cranberries and peanuts	tMCAO (I 2 h/R 24 h)	starting at 3 h after reperfusion and lasting for 4 days. I.P.	30 mg/kg	Caspase-3, Bcl2, Bax, MPO, TNF-α	neurological deficit, infarct volume, brain edema	Fang et al., 2015
Carvacrol	Lamiaceae	tMCAO (I 2 h/R 24 h)	1 and 12 h after the onset of MCAO. I.P.	10, 20, 40 mg/kg	MPO, TNF-α, IL-1β, iNOS, COX-2, SOD, MDA, NF-κB p65	Not detected	Li Y. et al., 2016
Curcumin	Curcuma aromatica Salisb.	pMCAO (24 h)	2 and 12 h after MCAO. I.P.	10, 50 mg/kg	MPO, TLR2, TLR4, NF-κB p65, TNF-α, IL-1β	neurological deficit, infarct volume, brain edema, Neutrophil Infiltration	Tu et al., 2014
Cannabidiol	Marijuana	tMCAO (I 4 h/R 24 h)	immediately before and 3 h after MCAO. I.P.	3 mg/kg	MPO, HMGB1	neurological deficit, infarct volume, Rota-rod test	Hayakawa et al., 2008
Allicin	garlic	tMCAO (I 1.5 h/R 24 h)	3 h after reperfusion daily for five consecutive days. I.P.	50 mg/kg	MPO, Caspase-3, TNF-α	neurological deficit, infarct volume, brain edema	Zhang et al., 2015
Ferulic acid	Angelica sinensis and Ligusticum chuanxiong	tMCAO (I 1.5 h/R 24 h)	Onset of MCAO, I.V.	100 mg/kg	ICAM-1, MPO, NF-κB	neurological deficit, infarct volume	Cheng et al., 2008
Ferulic acid	Angelica sinensis and Ligusticum chuanxiong	tMCAO (I 1.5 h/R 2 h)	Onset of MCAO, I.V.	100 mg/kg	ICAM-1, MPO, NF-κB	Not detected	Cheng et al., 2008
Rosmarinic acid	Rosmarinus officinalis	pMCAO	30 mins before and 1 h after pMCAO and lasting for 5 days. I.P.	20 mg/kg	MPO, SYP, BDNF	neurological deficit, infarct volume, Open-field test, Y-maze test, Object recognition test, Water maze test	Fonteles et al., 2016
Forsythoside B	Lamiophlomis rotata Kudo	tMCAO (I 1 h/R 23 h)	3, 5, 7 h after reperfusion. I.V.	20 mg/kg	NF-κB, IκB-α, MPO	neurological deficit, infarct volume, brain edema, BBB permeability	Jiang et al., 2010
Trigonelline	Fenugreek seeds	tMCAO (I 1.5 h/R 24 h)	30 min before MCAO, or immediately after MCAO or 1 h post MCAO. I.P.	100 mg/kg	MPO, GSH, MDA, Nitrite	neurological deficit, infarct volume	Pravalika et al., 2019
Dauricine	Menispermum dauricum DC	tMCAO (I 1 h/R 24 h)	1 h after MCAO. I.P.	5, 10 mg/kg	MPO, ICAM-1, TNF-α, IL-1β	Polymorphic neutrophils Infiltration	Yang X.Y. et al., 2007
Paeoniflorin	paeonia lactiflora Pall	tMCAO (I 1.5 h/R 24 h)	10 min before MCAO. I.V	20 mg/kg	MPO, TNF-α, IL-1β, ED1, ICAM-1, Apoptosis	neurological deficit, infarct volume	Tang N.Y. et al., 2010
β -Aescin	Aesculus hippocastanum	tMCAO (I 2 h/R 24 h)	Lasting for 7 days before MCAO. I.P.	60 mg/kg	ICAM-1, E-selectin, MPO	neurological deficit, infarct volume, Neutrophil Infiltration	Hu et al., 2004
Astragaloside IV	astragalus membranaceus	tMCAO (I 1.5 h/R 24 h)	immediately and 12 h after the onset of reperfusion. I.P.	10, 20 mg/kg	MPO, TNF-α, IL-1β, ICAM-1, NF-κB	neurological deficit, infarct volume	Li M. et al., 2012
Fucoidan	brown algae	tMCAO (I 2h/R24 h)	7 days before MCAO, I.P.	80, 160 mg/kg	IL-1β, IL-6, MPO, TNF-α, MDA, SOD, p-53, Bax, Bcl2, p-ERK, JNK, p38	neurological deficit, infarct volume	Che et al., 2017
Tanshinone IIA	Danshen	tMCAO (I 2 h/R 24 h)	10 min after MCAO, I.P.	25 mg/kg	MPO, MIF, TNF-α, IL-6	neurological deficit, infarct volume, brain edema	Chen et al., 2012
Osthole	Angelica pubescens, Cnidium monnieri and Peucedanum ostruthium	tMCAO (I 2 h/R 24 h)	30 min before MCAO, I.P.	20, 40 mg/kg	MDA, GSH, MPO, IL-1β, IL-8	neurological deficit, infarct volume, brain edema	Chao et al., 2010
(−)-α -bisabolol	Matricaria chamomilla	pMCAO	1 day before and 1 h after pMCAO and lasting for 5 days. I.G.	200 mg/kg	MPO, TNF-α, iNOS	neurological deficit, infarct volume, Open-field test, Y-maze test, Passive avoidance test, Object recognition test, Morris water maze	Fernandes et al., 2019
Sulforaphane	cruciferous vegetables	tMCAO (I 1 h/R 24 h)	the beginning of reper- fusion	5, 10 mg/kg	MPO, Caspase-1, IL-1β, IL-18, NLRP3	neurological deficit, infarct volume, neutrophils Infiltration	Yu et al., 2017

## Conclusion

In conclusion, MPO plays a vital role in mediating cerebral ischemia-reperfusion injury *via* mediating oxidative stress and neuroinflammation. Targeting MPO with natural compounds could be a promising strategy for treating ischemic stroke.

## Author Contributions

JS contributed as the senior author and the principal investigator (PI) of this study and refined the study. SC and HC wrote the first draft of the manuscript and contributed to the overall design. QD drew the structures of compounds. All authors read, critically reviewed, and approved the final manuscript.

## Conflict of Interest

The authors declare that the research was conducted in the absence of any commercial or financial relationships that could be construed as a potential conflict of interest.
